# High‐Resolution NGS HLA Typing Identifies Specific Class II–Dominant Risk Haplotypes and HLA LD Structure in Acute Lymphoblastic Leukaemia Among Ethnic Kazakhs

**DOI:** 10.1111/tan.70791

**Published:** 2026-06-09

**Authors:** Zhandos Burkitbayev, Aida Turganbekova, Zhulduz Zhanzakova, Dana Baimukasheva, Zhazira Saduakas, Didara Khamitova, Saniya Abdrakhmanova, Zhaksylyk Masalimov, Wassim Y. Almawi

**Affiliations:** ^1^ National Research Oncology Center Astana Kazakhstan; ^2^ Scientific and Production Center for Transfusiology Astana Kazakhstan; ^3^ Faculty of Natural Sciences L.N. Gumilyov Eurasian National University Astana Kazakhstan; ^4^ Faculty of Sciences El‐Manar University, El‐Manar University Campus at El‐Manar Tunis Tunisia

**Keywords:** acute lymphoblastic leukaemia, haplotypes, HLA polymorphisms, Kazakhstan, linkage disequilibrium, next‐generation sequencing

## Abstract

Acute lymphoblastic leukaemia (ALL) results from the uncontrolled growth of lymphoid progenitors, yet the immunogenetic factors behind this remain poorly understood. A connection between specific HLA alleles, multi‐locus haplotypes and ALL predisposition was suggested, with an ethnic component to this association. This retrospective case–control study analysed 137 ALL cases and 350 ethnically‐matched controls using six‐digit NGS typing of HLA‐A, ‐B, ‐C, ‐DRB1, ‐DQA1, ‐DQB1 and ‐DPB1 loci. Allele and haplotype differences were tested by *χ* ^2^/Fisher's exact methods with age‐ and sex‐adjusted logistic regression and Bonferroni/BH‐FDR correction. HLA haplotypes were generated using EM algorithms, and linkage disequilibrium (LD) was measured using ΔLD heatmaps. HLA Class I (C, B) and Class II (DRB1, DQA1, DQB1) alleles with strong, population‐specific links to ALL were identified in ethnic Kazakhs. Notable variants, including C*03:02:02, C*04:01:01, C*02:02:02, C*12:02:02, B*40:01:02 and B*27:05:02, as well as DRB1*14:54:01, DRB1*14:24:01 and DQB1*02:02:01, were found exclusively in cases. ΔLD analysis revealed structured, locus‐specific LD changes, with the most significant deviations involving DQA1/DQB1/DPB1 interactions. Class I haplotypes A*33:03:01~C*03:02:02~B*58:01:01 and A*33:03:01~C*03:02:01~B*58:01:01 showed positive and negative associations with ALL risk, respectively. Five Class II haplotypes, including DRB1*07:01:01~DQA1*01:02:01~DQB1*02:02:01~DPB1*04:01:01 and DRB1*15:01:01~ DQA1*05:05:01~DQB1*06:02:01~DPB1*04:02:01, were also strongly linked to a high ALL risk. In conclusion, NGS‐based high‐resolution typing identified novel, population‐specific HLA associations with ALL in ethnic Kazakhs, with Class II effects predominating and additional Class I signals at HLA‐B and HLA‐C. This provides the first detailed immunogenetic framework for ALL in Central Asia, highlighting the significance of population‐tailored HLA analyses for refining ALL risk assessment.

## 1 Introduction

Acute lymphoblastic leukaemia (ALL) is a haematological cancer characterised by the uncontrolled production of immature leukocytes in the blood and bone marrow and classified as B‐ALL (75%–80%) and T‐ALL (15%–25%) [1, 2]. ALL results from failures in controlling self‐renewal, maturation arrest at specific developmental stages, altered interactions between leukaemic blasts and the bone marrow microenvironment, and changes in HLA‐mediated antigen presentation, which may impair immune recognition and contribute to leukaemic progression [3, 4]. ALL pathogenesis is a stepwise process, starting in foetal haematopoiesis, in which prenatal abnormalities (mostly chromosomal hyperdiploidy or aneuploidy) drive the generation of clinically silent pre‐leukaemic populations [2, 5, 6]. ALL is the leading cancer in children [4], with a progressive rise in global prevalence [3, 7], and is a major contributor to cancer‐related deaths in paediatric patients [8]. While several genetic and immunogenetic factors associated with altered risk of ALL have been previously identified, the full range of inherited factors remains poorly understood [9, 10], the pathways by which immune imbalance facilitates the survival and leukemogenic capacity of pre‐leukaemic clones. Identifying these biomarkers and related mechanisms is key to accurate risk prediction and immune‐based therapies [9, 10].

The HLA system is a highly polymorphic multigene complex on chromosome 6 encoding Class I (HLA‐A, ‐C, ‐B) and Class II (HLA‐DR, ‐DQ, ‐DP) molecules, which are central to antigen presentation and immune recognition [11, 12]. Class I proteins present endogenous peptides to CD8^+^ T cells, while Class II heterodimers present exogenous peptides to CD4^+^ T cells [13, 14, 15]. Over 43,758 alleles are catalogued in the IPD‐IMGT/HLA Database, highlighting the extensive diversity across populations [12, 14]. Since earlier serological and PCR‐based assays cannot fully resolve this complexity, high‐resolution next‐generation sequencing (NGS) now offers precise four‐ and six‐digit allele discrimination and captures both coding and regulatory variants [13, 14]. This is crucial for defining population‐specific HLA patterns, reconstructing extended haplotypes and exploring how immunogenetic variation influences susceptibility to hematologic malignancies such as ALL. Given these roles, variation within the HLA region contributes to inter‐individual differences in cancer susceptibility.

Insofar as variation in the MHC region affects leukemogenesis [16, 17], several studies investigated the association between HLA variation and ALL susceptibility, reporting both risk and protective effects for specific alleles and haplotypes across different populations [18, 19]. Reported associations involving DPB1*02:01, DRB1*04:01, DQB1*05, and several Class I variants are most notable in East Asian populations [20, 21], but these signals vary across populations due to demographic history and MHC complex LD structure [16, 22]. Fine mapping uncovers multiple independent risk loci and changes in LD patterns [23, 24], while high‐resolution NGS now enables more precise analysis of these relationships [14, 25]. Findings were largely inconsistent due to differences in ethnic background, sample size and disease heterogeneity. As most studies focused on limited loci or specific subgroups, comprehensive analyses integrating both Class I and Class II variation remain relatively scarce. These limitations underscore the need for population‐specific and integrative analyses of HLA variation in ALL.

High‐resolution immunogenetic data from Central Asian populations, including Kazakhstan, remain limited compared with those from Europeans and East Asians [12, 14], an important gap given the population specificity of HLA allele frequencies and LD shaped by demographic history and selection [12, 16]. Kazakhstan, a Central Asian republic bridging Europe and Asia and its population of 20 million, comprises more than 130 ethnicities, of which ethnic Kazakhs account for 71.3%. Owing to their historical ties to European and Central/East Asian populations, ethnic Kazakhs possess HLA profiles that are distinct from those reported for European or East Asian populations [26, 27]. The unique Class II patterns of indigenous Kazakhs underscore the need for population‐specific analyses, but caution is warranted when extrapolating HLA‐disease associations across ethnic groups, as findings may not be directly transferable [26, 28]. In this context, ΔLD analyses help characterise haplotypic structures shaped by local evolutionary pressures within the MHC.

The present study investigates the association of HLA Class I and Class II alleles, haplotypes and LD patterns with ALL risk in a Kazakh cohort, aiming to identify population‐specific immunogenetic signatures. Because two‐field typing can obscure functionally relevant variants, six‐digit NGS provides the resolution needed for accurate inference of alleles, haplotypes and LD [14]. Using high‐resolution NGS in 137 ALL patients and 350 controls, we mapped allele frequencies, multi‐locus haplotypes and ΔLD structure, providing a high‐resolution immunogenetic characterisation of ALL in Kazakhstan. To our knowledge, this is among the first high‐resolution NGS‐based analyses of HLA variation in ALL in a Central Asian population.

## 2 Methods

### 2.1 Study Design and Outcomes

This was a retrospective, ethnically matched case–control association study. Immunophenotypic classification (B‐ALL vs. T‐ALL) was not available for all patients and was therefore not incorporated into the analysis. Alleles with frequencies < 1% in both cases and controls, or with cell counts < 5, were treated as rare and excluded from association testing to prevent unstable estimates. Such alleles were grouped or reported descriptively when appropriate, consistent with standard practice for highly polymorphic loci. The primary outcome was the ALL status, and primary exposures were high‐resolution six‐digit HLA alleles at HLA‐A, ‐B, ‐C, ‐DRB1, ‐DQA1, ‐DQB1 and ‐DPB1. Prespecified secondary analyses evaluated multi‐locus haplotypes (A~C~B and DRB1~DQA1~DQB1~DPB1) and LD structure (*r* ^2^) within and across loci, including differential LD (ΔLD) calculated as the difference between *r* ^2^_cases_ and *r* ^2^_controls_.

### 2.2 Study Subjects

We retrospectively identified 137 (self‐identified) ethnic Kazakh patients with newly diagnosed ALL who tested for HLA genotyping at the Research and Production Centre for Transfusiology (Astana, Kazakhstan) during December 2024–May 2025. Patients were recruited regardless of eligibility for haematopoietic stem cell transplantation and thus represent the general ALL population rather than a high‐risk subgroup. Age at diagnosis ranged from 4 to 56 years (mean ± SD: 21.0 ± 15.5 years). The cohort included 70 individuals (51.1%) aged 3–18 years, 47 (34.3%) aged 19–40 years and 20 (14.6%) aged ≥ 41 years. No age‐stratified analyses were performed. ALL diagnosis followed international standards, requiring ≥ 20% lymphoblasts on bone marrow morphology, lineage assignment by flow cytometry and cytogenetic or molecular confirmation when available. Only treatment‐naïve patients with adequate peripheral blood or bone marrow samples and complete clinical documentation were included.

Patients were excluded if they had prior hematologic malignancies, previous cytotoxic therapy, relapsed or refractory ALL, mixed‐phenotype or non‐lymphoid leukaemias, inadequate DNA quality, congenital immunodeficiencies, leukaemia‐predisposition syndromes, autoimmune disease requiring immunosuppression or active systemic infection. Age at diagnosis, sex, lineage classification (B‐ or T‐cell), and available cytogenetic or molecular risk markers, where available (including recurrent alterations such as BCR‐ABL1 and KMT2A rearrangements), were obtained from all participants. These were not included as covariates, and no exclusion or stratification by cytogenetic subtype was performed, as the primary objective was to assess germline HLA associations with overall ALL susceptibility. These descriptive variables were not incorporated into germline HLA association testing, as the analysis focused exclusively on inherited HLA variation and its relationship to overall ALL susceptibility.

The control group included 350 unrelated, self‐identified ethnic Kazakh stem cell donors who were collected within the same general timeframe (2023–2025) and the same national registry system as cases, minimising temporal and geographic sampling differences and who underwent NGS‐based HLA typing for Class I and II loci. Individuals of non‐Kazakh ancestry or with haematologic, autoimmune or chronic inflammatory disorders were excluded to limit population stratification. Both cases and controls were recruited from the same national population base and represent comparable ethnic and geographic backgrounds within Kazakhstan. Because donor registries may reflect healthy‐volunteer bias and haplotype enrichment, we evaluated representativeness by comparing control‐allele frequencies with published datasets from Kazakhs and Central Asians. Data on blast fraction in peripheral blood or bone marrow samples were not consistently available and were therefore excluded from the analysis. As a result, the potential influence of high blast burden on germline HLA inference cannot be entirely ruled out, although this risk is mitigated by sequencing depth and quality control procedures.

Reference datasets included published HLA frequency reports from Kazakh populations and regional cohorts, as well as publicly available data from international HLA databases [14, 26, 28], which showed that control‐group allele frequencies closely matched established population distributions, supporting the representativeness of the donor‐derived controls. The study was approved by the Local Bioethics Commission of the Scientific and Production Center for Transfusiology (SPCT/2024/6) and conducted in accordance with the Declaration of Helsinki.

### 2.3 DNA Extraction and HLA Typing

HLA typing for both ALL cases and controls was performed using the same workflow and setup at the Scientific and Production Center for Transfusiology (Astana, Kazakhstan). ALL cases and controls were processed using the MiSeq sequencing platform, the library preparation method and the HLA Twin v4.9.0 bioinformatics pipeline to ensure consistency and minimise technical bias. Total genomic DNA was extracted from participants' peripheral blood samples using the Inno‐Train Diagnostics automated BEX12 system (Kronberg im Taunus, Germany) with magnetic particle‐based separation and adjusted to 36 ng/μL using a Qubit fluorometer (Thermo Fisher Scientific, Almaty, Kazakhstan). High‐resolution HLA genotyping was done using Holotype HLA 24/7 kit (Omixon Inc., Hungary) with NGS for HLA‐A, ‐B, ‐C, ‐DRB1, ‐DQB1, ‐DQA1 and ‐DPB1. This allows for unambiguous allele assignment at high resolution, improving the detection of rare and closely related alleles, reducing the ambiguity inherent in lower‐resolution methods and enabling more accurate haplotype inference. Whole coding regions of the tested HLA loci, including the 5′ and 3′ untranslated regions, were amplified, with HLA‐DRB1 amplification targeting introns 1–4, while HLA‐DPB1 amplification included the UTR and exonic regions from introns 1–3.

Amplification was verified by agarose gel electrophoresis (2% w/v), and amplicon concentrations were measured using QuantiFluor dsDNA System (Promega, Madison, WI). Libraries were prepared through fragmentation, end‐repair and adapter ligation, then sequenced (600 μL, 9 pM) on the MiSeq platform (Illumina Inc., San Diego, CA). HLA typing was performed using HLA Twin software v4.9.0 (Omixon Inc., Budapest, Hungary) at six‐digit resolution, retaining only complete, validated profiles. Alleles were assigned using the IMGT/HLA database (https://www.ebi.ac.uk/ipd/imgt/hla/), Release 3.63.1 (February 2026). Quality metrics included locus‐specific call rates, read‐depth thresholds and ambiguity resolution, with low‐quality samples re‐sequenced or excluded. Because HLA typing was performed on peripheral blood collected at diagnosis, the presence of leukaemic blasts was considered. High‐depth sequencing (> 50× per exon), together with locus‐specific quality controls and allele balance assessment in the HLA Twin pipeline, minimises risks of allele dropout or misclassification due to somatic alterations such as LOH. No consistent evidence of allelic imbalance, monoallelic calls at normally heterozygous loci, or reduced call rates was observed, supporting accurate inference of germline HLA genotypes. The final dataset achieved call rates exceeding 99.5% across all loci.

### 2.4 Statistical Analysis

HLA Class I and II allele frequencies were obtained by direct counting, and case–control differences were tested using *χ* ^2^ or Fisher's exact test. Zero‐cell alleles were handled using the Haldane–Anscombe correction and verified with Firth regression; case–control counts were reported to assess sparse‐cell effects. Multiple‐testing adjustments were applied within loci, reflecting locus‐specific allele diversity and LD structure. A post hoc power analysis based on 137 cases and 350 controls (additive model, α = 0.05) estimated minimum detectable effects across allele‐frequency strata using standard R‐based methods, guiding interpretation of associations, particularly for low‐frequency variants. Sensitivity analyses excluding extremely rare alleles (frequency < 1%) yielded consistent signals. Odds ratios (ORs) with 95% confidence intervals (CIs) were generated using logistic regression, with age and sex included as non‐significant covariates for completeness. Rare (< 1%) and group‐exclusive alleles were interpreted cautiously, as sparse data can yield unstable estimates even after Haldane–Anscombe correction and Firth regression. Given the high polymorphism and LD in HLA, both Bonferroni and Benjamini–Hochberg FDR adjustments were applied, the Bonferroni correction, determined as: $p_{c}=1-{\left(1-p_{\mathrm{adj}}\right)}^{n}$, where *n* represents the number of alleles tested within the given locus. Bonferroni‐significant results were treated as robust, whereas associations detected only by FDR were considered suggestive, and thus interpreted with caution. Fisher residual deviation assessed allele‐level deviations under conditions of high polymorphism and sparse cell counts. Hardy–Weinberg equilibrium (HWE) in controls was tested using Monte Carlo exact tests with 10,000 replicates to detect genotyping errors or hidden structure; consistent Class II results provided no evidence of Wahlund effects. For alleles with zero or very low counts, Firth's penalised logistic regression was used to minimise small‐sample bias, and continuity‐corrected ORs were reported as conservative, preliminary estimates that require further validation.

Linkage disequilibrium was estimated from unphased genotypes using a composite multiallelic *r* ^2^ derived from EM‐estimated haplotype frequencies, which extends the squared correlation coefficient to multi‐allelic loci by incorporating the full haplotype distribution. Pairwise *r* ^2^ values were used to generate locus‐level LD heatmaps separately for cases and controls, and differential LD was summarised as ΔLD, defined as the difference between composite multiallelic *r* ^2^ values in cases and controls (ΔLD = *r* ^2^_cases_ − *r* ^2^_controls_) to highlight locus pairs with altered haplotype structure in the disease group. All LD heatmaps and ΔLD visualisations presented in the figures are based on this multiallelic *r* ^2^ metric, which captures shifts in non‐random allele association across this highly polymorphic region. D′ was calculated as a secondary measure and reported in Figure S1. LD estimates were interpreted cautiously, given the difference in sample sizes between cases and controls, as smaller sample sizes may affect haplotype diversity and inflate LD estimates. Class I three‐locus and Class II four‐locus haplotypes were inferred with expectation–maximization (EM) in Arlequin 3.5.2.2, which yields stable frequency estimates for common combinations but is less reliable for low‐frequency haplotypes in highly polymorphic regions and moderate sample sizes. Estimation uncertainty for these rare combinations was therefore acknowledged in the interpretation, and haplotype associations were evaluated using *χ* ^2^ or Fisher's exact tests and logistic regression with Bonferroni and BH‐FDR corrections. As low‐frequency haplotypes produced imprecise estimates with wide CIs, this reflects limited power in a highly polymorphic region. To assess stability and reduce sparse‐cell influence, association analyses were repeated after restricting to alleles with frequencies ≥ 1% in cases or controls. All analyses were conducted in R 4.3.0, and statistical significance was evaluated at both nominal (*p* < 0.05) and multiple‐testing‐corrected levels, with only Bonferroni‐ or FDR‐adjusted associations considered robust and nominal findings treated as exploratory; significance was defined as two‐tailed *p* < 0.05.

## 3 Results

The ALL cohort included a mix of patients, as described in the Section 2, and associations were analysed across the entire cohort without stratification by immunophenotypic subtype. The cohort was not restricted to patients eligible for haematopoietic stem cell transplantation. Allele frequency distributions for HLA Class I (A, C, B) and Class II (DRB1, DQA1, DQB1, DPB1) loci were assessed in 137 ALL cases and 350 controls. *χ* ^2^ tests with age‐ and sex‐adjusted *p* values, ORs and 95% CIs were applied, with Bonferroni and FDR corrections. The number of alleles tested varied by locus, and multiple testing correction was applied accordingly using locus‐specific Bonferroni adjustments. Post hoc power analysis indicated that the study was adequately powered (> 80%) to detect moderate effect sizes (OR = 1.8–2.2) for common alleles, whereas substantially larger effects were required for intermediate and rare variants. Alleles with frequency ≥ 1% were considered ‘common’, whereas those < 1% were classified as ‘rare’. Monte Carlo simulation (10,000 replicates) showed no significant deviation for HLA‐A, ‐B, ‐C, DRB1 and DQB1, while modest deviations were observed for DQA1 (*p* = 0.039) and DPB1 (*p* = 0.006) (Table S1), likely arising from the complexity of allele frequency distributions, limited sample size or subtle population substructure rather than genotyping error. Several alleles were seen exclusively in either cases or controls, resulting in large ORs driven by zero or near‐zero cell counts, reflecting sparse‐cell effects and should therefore be interpreted cautiously (Tables 1, 2, 3, 4, 5, 6, 7). The nominal deviations at DQA1 and DPB1 prompted prespecified sensitivity analyses restricted to alleles ≥ 1% frequency and excluding sparse categories; signal directionality remained consistent.

**TABLE 1:** HLA‐A* allele distribution among acute lymphoblastic leukaemia cases and controls^a^.

Allele	Cases^b^	Controls^b^	Chi‐square	*p* ^c^	OR (95% CI)^c^	Corrected *p* ^d^	FDR *p* ^e^
31:01:01	0 (0.00000)	32 (0.04570)	12.951	< 0.0001	0.037 (0.002–0.614)	0.006	0.006
68:01:01	2 (0.00730)	26 (0.03710)	6.282	0.012	0.191 (0.045–0.809)	0.208	0.116
25:01:01	7 (0.02550)	6 (0.00860)	4.309	0.038	3.032 (1.010–9.106)	0.520	0.240
32:01:01	4 (0.01460)	24 (0.03430)	2.733	0.098	0.417 (0.143–1.214)	0.860	0.405
33:03:01	22 (0.08030)	37 (0.05290)	2.605	0.107	1.564 (0.905–2.704)	0.882	0.405
23:01:01	2 (0.00730)	13 (0.01860)	1.650	0.199	0.389 (0.087–1.733)	0.985	0.472
30:01:01	9 (0.03280)	13 (0.01860)	1.818	0.178	1.795 (0.758–4.248)	0.976	0.472
11:01:01	17 (0.06200)	62 (0.08860)	1.859	0.173	0.681 (0.390–1.187)	0.973	0.472
02:07:01	5 (0.01820)	20 (0.02860)	0.839	0.360	0.632 (0.235–1.701)	1.000	0.633
01:01:01	19 (0.06930)	60 (0.08570)	0.708	0.400	0.795 (0.465–1.358)	1.000	0.633
02:01:01	5 (0.20070)	124 (0.17710)	0.730	0.393	1.167 (0.819–1.661)	1.000	0.633
29:01:01	2 (0.00730)	11 (0.01570)	1.059	0.303	0.461 (0.101–2.091)	0.999	0.633
03:01:01	14 (0.05110)	44 (0.06290)	0.486	0.486	0.803 (0.433–1.490)	1.000	0.710
03:02:01	1 (0.00360)	5 (0.00710)	0.393	0.531	0.509 (0.059–4.378)	1.000	0.721
02:05:01	2 (0.00730)	7 (0.01000)	0.157	0.692	0.728 (0.150–3.526)	1.000	0.853
24:02:01	54 (0.19710)	132 (0.18860)	0.092	0.761	1.056 (0.742–1.503)	1.000	0.853
26:01:01	15 (0.05470)	35 (0.05000)	0.091	0.763	1.100 (0.591–2.049)	1.000	0.853
02:06:01	10 (0.03650)	24 (0.03430)	0.029	0.866	1.067 (0.503–2.262)	1.000	0.914
66:01:01	2 (0.00730)	5 (0.00710)	0.001	0.979	1.022 (0.197–5.300)	1.000	0.979

^a^ Study subjects comprised 137 ALL cases and 350 controls.

^b^ Allele count (frequency).

^c^ Adjusted for age and gender.

^d^ Denotes the Bonferroni‐adjusted age‐ and sex‐adjusted *p* value derived from logistic regression, calculated as: $p_{c}=1-{\left(1-p_{\mathrm{adj}}\right)}^{n}$, where n is the number of alleles tested at that locus.

^e^ Associations surviving both Bonferroni and FDR correction were considered robust, whereas those significant only under FDR were treated as suggestive due to its less stringent error control.

**TABLE 2:** HLA‐C* Allele distribution among acute lymphoblastic leukaemia cases and controls^a^.

Allele	Cases^b^	Controls^b^	Chi‐square	*p* ^c^	OR (95% CI)^c^	Corrected *p* ^d^	FDR *p* ^e^
03:02:02	19 (0.06930)	0 (0.00000)	49.506	< 0.001	106.926 (6.432–1777.488)	< 0.001	< 0.001
04:01:01	19 (0.06930)	0 (0.00000)	49.506	< 0.001	106.926 (6.432–1777.488)	< 0.001	< 0.001
04:01:02	0 (0.00000)	75 (0.10710)	31.806	< 0.001	0.015 (0.001–0.244)	< 0.001	< 0.001
02:02:02	8 (0.02920)	0 (0.00000)	20.607	< 0.001	44.685 (2.570–776.921)	< 0.001	< 0.001
12:02:02	7 (0.02550)	0 (0.00000)	18.013	< 0.001	39.280 (2.236–690.180)	< 0.001	< 0.001
03:02:01	0 (0.00000)	38 (0.05430)	15.478	< 0.001	0.031 (0.002–0.512)	0.002	< 0.001
02:02:01	0 (0.00000)	22 (0.03140)	8.810	0.003	0.055 (0.003–0.909)	0.064	0.009
12:02:01	1 (0.00360)	25 (0.03570)	7.793	0.005	0.099 (0.013–0.734)	0.109	0.014
12:03:01	14 (0.05110)	50 (0.07140)	1.326	0.249	0.700 (0.380–1.288)	0.998	0.594
14:02:01	10 (0.03650)	17 (0.02430)	1.089	0.297	1.522 (0.688–3.366)	1.000	0.594
01:02:01	15 (0.05470)	51 (0.07290)	1.023	0.312	0.737 (0.407–1.334)	1.000	0.594
03:03:01	14 (0.05110)	26 (0.03710)	0.973	0.324	1.396 (0.718–2.715)	1.000	0.594
07:01:01	11 (0.04010)	38 (0.05430)	0.824	0.364	0.729 (0.367–1.447)	1.000	0.616
03:04:01	27 (0.09850)	58 (0.08290)	0.608	0.436	1.210 (0.749–1.955)	1.000	0.669
07:04:01	9 (0.03280)	17 (0.02430)	0.555	0.456	1.364 (0.601–3.099)	1.000	0.669
06:02:01	38 (0.13870)	87 (0.12430)	0.365	0.546	1.135 (0.753–1.709)	1.000	0.750
15:02:01	17 (0.06200)	40 (0.05710)	0.086	0.770	1.091 (0.608–1.960)	1.000	0.949
07:02:01	27 (0.09850)	72 (0.10290)	0.040	0.841	0.953 (0.598–1.520)	1.000	0.949
08:01:01	7 (0.02550)	19 (0.02710)	0.019	0.890	0.940 (0.391–2.261)	1.000	0.949
08:02:01	4 (0.01460)	11 (0.01570)	0.016	0.899	0.928 (0.293–2.939)	1.000	0.949
05:01:01	6 (0.02190)	16 (0.02290)	0.008	0.928	0.957 (0.371–2.472)	1.000	0.949
08:03:01	3 (0.01090)	8 (0.01140)	0.004	0.949	0.958 (0.252–3.636)	1.000	0.949

^a^ Study subjects comprised 137 ALL cases and 350 controls.

^b^ Allele count (frequency).

^c^ Adjusted for age and gender.

^d^ Denotes the Bonferroni‐adjusted age‐ and sex‐adjusted *p* value derived from logistic regression, calculated as: $p_{c}=1-{\left(1-p_{\mathrm{adj}}\right)}^{n}$, where n is the number of alleles tested at that locus.

^e^ Associations surviving both Bonferroni and FDR correction were considered robust, whereas those significant only under FDR were treated as suggestive due to their less stringent error control.

**TABLE 3:** HLA‐B* allele distribution among acute lymphoblastic leukaemia cases and controls^a^.

Allele	Cases^b^	Controls^b^	Chi‐square	*p* ^c^	OR (95% CI)^c^	Corrected *p* ^d^	FDR *p* ^e^
01:01:01	0 (0.00000)	114 (0.16290)	50.538	< 0.0001	0.009 (0.001–0.151)	< 0.0001	< 0.0001
51:01:01	25 (0.09120)	11 (0.01570)	31.558	< 0.0001	6.289 (3.049–12.969)	< 0.0001	< 0.0001
40:01:02	7 (0.02550)	0 (0.00000)	18.013	< 0.0001	39.280 (2.236–690.180)	0.001	< 0.0001
27:05:02	5 (0.01820)	0 (0.00000)	12.840	< 0.0001	28.592 (1.576–518.863)	0.011	0.003
44:02:01	10 (0.03650)	5 (0.00710)	11.190	0.001	5.265 (1.783–15.548)	0.026	0.005
57:01:01	5 (0.01820)	1 (0.00140)	9.099	0.003	12.993 (1.511–111.726)	0.079	0.013
52:01:01	8 (0.02920)	4 (0.00570)	8.924	0.003	5.233 (1.563–17.523)	0.086	0.013
27:05:01	0 (0.00000)	19 (0.02710)	7.585	0.006	0.064 (0.004–1.058)	0.172	0.024
44:03:01	5 (0.01820)	2 (0.00290)	6.538	0.011	6.487 (1.251–33.639)	0.288	0.038
41:02:01	0 (0.00000)	15 (0.02140)	5.963	0.015	0.081 (0.005–1.351)	0.376	0.047
20:03:01	0 (0.00000)	13 (0.01860)	5.157	0.023	0.093 (0.005–1.566)	0.527	0.065
50:01:01	6 (0.02190)	4 (0.00570)	5.075	0.024	3.896 (1.091–13.913)	0.544	0.065
16:01:01	0 (0.00000)	10 (0.01430)	3.955	0.047	0.120 (0.007–2.051)	0.784	0.115
37:01:01	6 (0.02190)	7 (0.01000)	2.117	0.146	2.216 (0.738–6.655)	0.994	0.297
35:03:01	7 (0.02550)	32 (0.04570)	2.084	0.149	0.547 (0.239–1.255)	0.994	0.297
46:01:01	3 (0.01090)	18 (0.02570)	2.035	0.154	0.419 (0.123–1.435)	0.995	0.297
14:01:01	1 (0.00360)	10 (0.01430)	1.995	0.158	0.253 (0.032–1.984)	0.996	0.297
08:01:01	6 (0.02190)	26 (0.03710)	1.440	0.230	0.580 (0.236–1.426)	1.000	0.409
58:01:01	22 (0.08030)	42 (0.06000)	1.321	0.250	1.368 (0.800–2.337)	1.000	0.418
35:02:01	4 (0.01460)	5 (0.00710)	1.196	0.274	2.059 (0.549–7.726)	1.000	0.418
55:02:01	4 (0.01460)	5 (0.00710)	1.196	0.274	2.059 (0.549–7.726)	1.000	0.418
18:01:01	12 (0.04380)	22 (0.03140)	0.894	0.344	1.412 (0.689–2.893)	1.000	0.500
48:01:01	5 (0.01820)	20 (0.02860)	0.839	0.360	0.632 (0.235–1.701)	1.000	0.500
15:01:01	6 (0.02190)	22 (0.03140)	0.641	0.423	0.690 (0.277–1.720)	1.000	0.565
38:01:01	5 (0.01820)	9 (0.01290)	0.404	0.525	1.427 (0.474–4.297)	1.000	0.638
40:02:01	17 (0.06200)	37 (0.05290)	0.317	0.573	1.185 (0.656–2.143)	1.000	0.638
13:01:01	3 (0.01090)	11 (0.01570)	0.316	0.574	0.693 (0.192–2.505)	1.000	0.638
14:02:01	3 (0.01090)	11 (0.01570)	0.316	0.574	0.693 (0.192–2.505)	1.000	0.638
13:02:01	22 (0.08030)	49 (0.07000)	0.309	0.579	1.160 (0.687–1.958)	1.000	0.638
07:02:01	18 (0.06570)	40 (0.05710)	0.257	0.612	1.160 (0.653–2.061)	1.000	0.653
35:01:01	16 (0.05840)	36 (0.05140)	0.189	0.664	1.144 (0.624–2.097)	1.000	0.685
15:18:01	5 (0.01820)	15 (0.02140)	0.099	0.753	0.849 (0.305–2.358)	1.000	0.753

^a^ Study subjects comprised 137 ALL cases and 350 controls.

^b^ Allele count (frequency).

^c^ Adjusted for age and gender.

^d^ Denotes the Bonferroni‐adjusted age‐ and sex‐adjusted *p* value derived from logistic regression, calculated as: $p_{c}=1-{\left(1-p_{\mathrm{adj}}\right)}^{n}$, where n is the number of alleles tested at that locus.

^e^ Associations surviving both Bonferroni and FDR correction were considered robust, whereas those significant only under FDR were treated as suggestive due to their less stringent error control.

**TABLE 4:** HLA‐DRB1* allele distribution among acute lymphoblastic leukaemia cases and controls^a^.

Allele	Cases^b^	Controls^b^	Chi‐square	*p* ^c^	OR (95% CI)^c^	Corrected *p* ^d^	FDR *p* ^e^
14:54:01	5 (0.01820)	0 (0.00000)	12.840	< 0.0001	28.592 (1.576–518.863)	0.007	0.004
14:01:01	0 (0.00000)	31 (0.04430)	12.533	< 0.0001	0.039 (0.002–0.635)	0.009	0.004
14:24:01	4 (0.01460)	0 (0.00000)	10.261	0.001	23.307 (1.251–434.375)	0.029	0.010
11:04:01	13 (0.04740)	15 (0.02140)	4.774	0.029	2.275 (1.068–4.846)	0.475	0.159
04:03:01	1 (0.00360)	15 (0.02140)	3.852	0.050	0.167 (0.022–1.273)	0.674	0.219
14:12:01	4 (0.01460)	3 (0.00430)	2.935	0.087	3.442 (0.765–15.481)	0.864	0.318
04:02:01	4 (0.01460)	4 (0.00570)	1.908	0.167	2.578 (0.640–10.381)	0.982	0.501
15:02:01	13 (0.04740)	21 (0.03000)	1.779	0.182	1.610 (0.795–3.263)	0.988	0.501
04:01:01	18 (0.06570)	61 (0.08710)	1.216	0.270	0.737 (0.427–1.271)	0.999	0.661
12:01:01	12 (0.04380)	24 (0.03430)	0.500	0.479	1.290 (0.636–2.617)	1.000	0.868
01:01:01	17 (0.06200)	52 (0.07430)	0.448	0.503	0.824 (0.468–1.452)	1.000	0.868
08:03:02	6 (0.02190)	11 (0.01570)	0.439	0.508	1.402 (0.513–3.830)	1.000	0.868
03:01:01	24 (0.08760)	71 (0.10140)	0.428	0.513	0.850 (0.523–1.382)	1.000	0.868
07:01:01	37 (0.13500)	89 (0.12710)	0.109	0.741	1.072 (0.710–1.618)	1.000	0.999
13:01:01	14 (0.05110)	39 (0.05570)	0.082	0.775	0.913 (0.487–1.709)	1.000	0.999
04:04:01	4 (0.01460)	12 (0.01710)	0.079	0.779	0.849 (0.272–2.657)	1.000	0.999
10:01:01	4 (0.01460)	12 (0.01710)	0.079	0.779	0.849 (0.272–2.657)	1.000	0.999
15:01:01	25 (0.09120)	67 (0.09570)	0.046	0.830	0.949 (0.586–1.536)	1.000	0.999
12:02:01	6 (0.02190)	16 (0.02290)	0.008	0.928	0.957 (0.371–2.472)	1.000	0.999
13:02:01	10 (0.03650)	26 (0.03710)	0.002	0.962	0.982 (0.467–2.064)	1.000	0.999
11:01:01	11 (0.04010)	28 (0.04000)	0.000	0.992	1.004 (0.493–2.046)	1.000	0.999
09:01:02	9 (0.03280)	23 (0.03290)	0.000	0.999	1.000 (0.457–2.189)	1.000	0.999

^a^ Study subjects comprised 137 ALL cases and 350 controls.

^b^ Allele count (frequency).

^c^ Adjusted for age and gender.

^d^ Denotes the Bonferroni‐adjusted age‐ and sex‐adjusted *p* value derived from logistic regression, calculated as: $p_{c}=1-{\left(1-p_{\mathrm{adj}}\right)}^{n}$, where n is the number of alleles tested at that locus.

^e^ FDR = false discovery rate, calculated according to Benjamini–Hochberg (BH) Procedure. Associations surviving both Bonferroni and FDR correction were considered robust, whereas those significant only under FDR were treated as suggestive due to their less stringent error control.

**TABLE 5:** HLA‐DQA1* allele distribution among acute lymphoblastic leukaemia cases and controls^a^.

Allele	Cases^b^	Controls^b^	Chi‐square	*p* ^c^	OR (95% CI)^c^	Corrected *p* ^d^	FDR *p* ^e^
05:05:01	32 (0.12900)	0 (0.00000)	93.478	< 0.0001	210.312 (12.824–3449.020)	< 0.0001	< 0.0001
03:03:01	19 (0.07660)	0 (0.00000)	54.726	< 0.0001	119.039 (7.159–1979.459)	< 0.0001	< 0.0001
01:04:01	10 (0.04030)	0 (0.00000)	28.527	< 0.0001	61.679 (3.600–1056.628)	< 0.0001	< 0.0001
03:02:01	10 (0.04030)	0 (0.00000)	28.527	< 0.0001	61.679 (3.600–1056.628)	< 0.0001	< 0.0001
03:01:01	16 (0.06450)	146 (0.20860)	26.821	< 0.0001	0.262 (0.153–0.448)	< 0.0001	< 0.0001
05:03:01	7 (0.02820)	0 (0.00000)	19.905	< 0.0001	43.509 (2.476–764.691)	< 0.0001	< 0.0001
01:01:01	14 (0.05650)	110 (0.15710)	16.330	< 0.0001	0.321 (0.180–0.571)	0.001	< 0.0001
05:01:01	26 (0.10480)	155 (0.22140)	16.114	< 0.0001	0.412 (0.264–0.642)	0.001	< 0.0001
04:01:01	4 (0.01610)	7 (0.01000)	0.600	0.439	1.623 (0.471–5.592)	0.999	0.634
02:01:01	35 (0.14110)	89 (0.12710)	0.315	0.575	1.128 (0.740–1.719)	1.000	0.747
01:03:01	26 (0.11690)	78 (0.11140)	0.056	0.814	1.056 (0.671–1.661)	1.000	0.872
06:01:01	7 (0.02820)	18 (0.02570)	0.045	0.832	1.101 (0.454–2.667)	1.000	0.872
01:02:01	33 (0.13310)	96 (0.13710)	0.026	0.872	0.966 (0.631–1.477)	1.000	0.872

^a^ Allele count (frequency).

^b^ Crude (unadjusted) analysis.

^c^ Adjusted for age and gender.

^d^ Denotes the Bonferroni‐adjusted age‐ and sex‐adjusted *p* value derived from logistic regression, calculated as: $p_{c}=1-{\left(1-p_{\mathrm{adj}}\right)}^{n}$, where n is the number of alleles tested at that locus.

^e^ FDR = false discovery rate, calculated according to Benjamini–Hochberg (BH) Procedure. Associations surviving both Bonferroni and FDR correction were considered robust, whereas those significant only under FDR were treated as suggestive due to their less stringent error control.

**TABLE 6:** HLA‐DQB1* allele distribution among acute lymphoblastic leukaemia cases and controls^a^.

Allele	Cases^b^	Controls^b^	Chi‐square	*p* ^c^	OR (95% CI)^c^	Corrected *p* ^d^	FDR *p* ^e^
02:02:01	30 (0.10950)	0 (0.00000)	79.078	< 0.0001	174.767 (10.646–2869.030)	< 0.0001	< 0.0001
02:01:01	28 (0.10220)	148 (0.21140)	15.873	< 0.0001	0.425 (0.276–0.653)	0.001	0.001
05:03:01	4 (0.01460)	28 (0.04000)	3.999	0.046	0.356 (0.124–1.023)	0.503	0.228
04:01:01	1 (0.00360)	10 (0.01430)	1.995	0.158	0.253 (0.032–1.984)	0.924	0.592
03:01:01	69 (0.25180)	152 (0.21710)	1.350	0.245	1.213 (0.875–1.682)	0.985	0.736
04:02:01	6 (0.02190)	10 (0.01430)	0.706	0.401	1.545 (0.556–4.292)	1.000	0.943
05:02:01	11 (0.04010)	22 (0.03140)	0.457	0.499	1.289 (0.616–2.695)	1.000	0.943
05:01:01	23 (0.08390)	67 (0.09570)	0.325	0.568	0.866 (0.527–1.421)	1.000	0.943
06:09:01	4 (0.01460)	8 (0.01140)	0.163	0.687	1.281 (0.383–4.291)	1.000	0.943
03:02:01	20 (0.07300)	56 (0.08000)	0.134	0.714	0.906 (0.533–1.540)	1.000	0.943
06:04:01	6 (0.02190)	18 (0.02570)	0.119	0.730	0.848 (0.333–2.160)	1.000	0.943
06:02:01	24 (0.08760)	57 (0.08140)	0.098	0.754	1.083 (0.658–1.783)	1.000	0.943
06:03:01	16 (0.05840)	39 (0.05570)	0.026	0.871	1.051 (0.577–1.914)	1.000	0.961
03:03:02	12 (0.04380)	32 (0.04570)	0.017	0.897	0.956 (0.485–1.885)	1.000	0.961
06:01:01	17 (0.06200)	43 (0.06140)	0.001	0.971	1.011 (0.566–1.805)	1.000	0.971

^a^ Allele count (frequency).

^b^ Crude (unadjusted) analysis.

^c^ Adjusted for age and gender.

^d^ Denotes the Bonferroni‐adjusted age‐ and sex‐adjusted *p* value derived from logistic regression, calculated as: $p_{c}=1-{\left(1-p_{\mathrm{adj}}\right)}^{n}$, where n is the number of alleles tested at that locus.

^e^ FDR = false discovery rate, calculated according to Benjamini–Hochberg (BH) Procedure. Associations surviving both Bonferroni and FDR correction were considered robust, whereas those significant only under FDR were treated as suggestive due to their less stringent error control.

**TABLE 7:** HLA‐DPB1* allele distribution among acute lymphoblastic leukaemia cases and controls^a^.

Allele	Cases^b^	Controls^b^	Chi‐square	*p* ^c^	OR (95% CI)^c^	Corrected *p* ^d^	FDR *p* ^e^
04:01:01	99 (0.37500)	229 (0.32710)	1.956	0.162	1.234 (0.919–1.657)	0.899	0.829
02:01:02	38 (0.14390)	125 (0.17860)	1.637	0.201	0.773 (0.521–1.147)	0.946	0.829
01:01:01	1 (0.00380)	9 (0.01290)	1.536	0.215	0.292 (0.037–2.316)	0.957	0.829
02:02:01	2 (0.00760)	10 (0.01430)	0.702	0.402	0.527 (0.115–2.420)	0.999	0.829
14:01:01	3 (0.01140)	13 (0.01860)	0.610	0.435	0.607 (0.172–2.149)	0.999	0.829
03:01:01	20 (0.07580)	64 (0.09140)	0.592	0.442	0.815 (0.483–1.375)	0.999	0.829
09:01:01	4 (0.01520)	16 (0.02290)	0.560	0.454	0.658 (0.218–1.986)	1.000	0.829
13:01:01	6 (0.02270)	21 (0.03000)	0.372	0.542	0.752 (0.300–1.884)	1.000	0.829
15:01:01	2 (0.00760)	8 (0.01140)	0.277	0.599	0.660 (0.139–3.130)	1.000	0.829
05:01:01	22 (0.08330)	52 (0.07430)	0.221	0.638	1.133 (0.674–1.905)	1.000	0.829
17:01:01	10 (0.03790)	28 (0.04000)	0.023	0.880	0.945 (0.452–1.973)	1.000	0.920
04:02:01	33 (0.12500)	90 (0.12860)	0.022	0.882	0.968 (0.632–1.483)	1.000	0.920
10:01:01	4 (0.01520)	10 (0.01430)	0.010	0.920	1.062 (0.330–3.414)	1.000	0.920

^a^ Allele count (frequency).

^b^ Crude (unadjusted) analysis.

^c^ Adjusted for age and gender.

^d^ Denotes the Bonferroni‐adjusted age‐ and sex‐adjusted *p* value derived from logistic regression, calculated as: $p_{c}=1-{\left(1-p_{\mathrm{adj}}\right)}^{n}$, where n is the number of alleles tested at that locus.

^e^ FDR = false discovery rate, calculated according to Benjamini–Hochberg (BH) Procedure. Associations surviving both Bonferroni and FDR correction were considered robust, whereas those significant only under FDR were treated as suggestive due to their less stringent error control.

Although several associations remained significant after multiple‐testing correction, estimates involving low‐frequency alleles require caution, as sparse data can inflate effect sizes. Rare alleles were excluded from formal testing and described only when informative. Zero‐cell comparisons, yielding large ORs, were handled with Haldane–Anscombe correction and confirmed with Firth regression, with case–control counts reported to reflect uncertainty. Associations are classified as robust when significant under both Bonferroni and FDR, and suggestive when FDR‐only. Cytogenetic and molecular subgrouping was not feasible, but control frequencies aligned with published Kazakh and regional datasets, supporting the validity of comparisons. No systematic genotyping anomalies were observed in cases, including unexpected homozygosity across multiple loci or reduced call rates, thereby arguing against widespread effects of somatic LOH on HLA typing.

### 3.1 HLA Class I Allele Distribution

A total of 36 HLA‐A alleles were observed, of which 17 were common (frequencies > 1.0%), and the remaining 19 were considered rare (frequencies < 1.0%), Of the common A alleles, A*31:01:01 (*n* = 0/137 vs. 32/350) demonstrated a robust association (corrected *p* = 0.006, FDR *p* = 0.006), and was absent in cases but present in controls [OR (95% CI) = 0.037 (0.0002–0.614)], indicating a potential protective effect (Table 1). A*31:01:01 was reported in Central Asian and East Asian populations at moderate frequencies, suggesting that its apparent protective association may reflect both population background and disease‐related depletion in cases. Marginal associations were also noted for A*68:01:01 (*n* = 2/137 vs. 262/350), which was under‐represented in cases than controls [*p* = 0.012; OR (95% CI) = 0.191 (0.045–0.809)], and A*25:01:01 (*n* = 7/137 vs. 6/350), which was enriched in cases [*p* = 0.038; OR (95% CI) = 3.032 (1.010–9.106)]. However, the significance of these associations was lost after correcting for multiple testing. The allele frequencies of the remaining HLA‐A alleles were largely comparable between cases and controls (Table 1).

We identified 42 HLA‐C alleles in this cohort. Of these, 20 were common (frequency > 1.0%), while the remaining 22 were rare/uncommon (frequency < 1.0%). Significant heterogeneity was observed at the HLA‐C locus, notably with some HLA‐C alleles present only in one group. These comprised C*03:02:02 (*n* = 19/137 vs. 0/350; OR = 106.926), C*04:01:01 (*n* = 19/137 vs. 0/350; OR = 106.926), C*02:02:02 (*n* = 8/137 vs. 0/350; OR = 44.685) and C*12:02:02 (*n* = 7/137 vs. 0/350; OR = 39.280), which were exclusively present in cases (all FDR *p* < 0.001). In contrast, C*04:01:02 (*n* = 0/137 vs. 75/350; OR = 0.015; FDR *p* < 0.001), C*03:02:01 (*n* = 0/137 vs. 38/350; OR = 0.031; FDR *p* < 0.001) and C*02:02:01 (*n* = 0/137 vs. 22/350; OR = 0.055; FDR *p* = 0.009) were found only in controls and thus associated with reduced risk of ALL. C*04:01:02 and C*03:02:01 are known to occur in regional populations, suggesting that their absence in cases may reflect disease‐related under‐representation rather than population‐specificity. The initially negative associations of C*12:02:01 persisted after FDR correction (FDR *p* < 0.001) but not after Bonferroni adjustment (Corrected *p* = 0.109) (Table 2), indicating a suggestive association that does not meet the more stringent threshold for family‐wise error control and should therefore be interpreted cautiously. Other alleles identified showed no significant link to an altered ALL risk. These unusually extreme effect sizes reflect sparse‐cell counts, and the absence of these alleles in controls rather than precise estimates of biological magnitude should not be overinterpreted.

The HLA‐B locus exhibited the greatest allelic diversity, with 73 distinct alleles identified, of which 28 were common alleles (frequencies > 1.0%). Significantly higher prevalence of B*40:01:02 (*n* = 79/137 vs. 0/350; OR = 39.280; FDR *p* < 0.001) and B*27:05:02 (*n* = 5/137 vs. 0/350; OR = 28.592; FDR *p* = 0.003) (exclusively found in patients), B*51:01:01 (*n* = 25/137 vs. 11/350; OR = 6.289; FDR *p* < 0.001) and B*44:02:01 (*n* = 10/137 vs. 5/350; OR = 5.265; FDR *p* = 0.005) were seen in ALL cases than in controls (Table 3). On the other hand, B*01:01:01 (*n* = 0/137 vs. 114/350; OR = 0.009; FDR *p* < 0.001) (absent in cases), B*27:05:01 (*n* = 0/137 vs. 19/350; OR = 0.064; FDR *p* = 0.024) and B*41:02:01 (*n* = 0/137 vs. 15/350; OR = 0.081; FDR *p* = 0.047) were more common in controls, suggesting protection. Furthermore, the initial significant associations of B*20:03:01 (*p* = 0.023) and B*16:01:01 (p = 0.047) were lost after correction. Collectively, this reflects a non‐random, biologically meaningful distribution of HLA‐B alleles with respect to ALL risk.

### 3.2 HLA Class II Allele Distribution

In total, 49 HLA‐DRB1 alleles were identified, of which 22 were common (frequencies > 1.0%), with both positive and negative associations seen after adjusting for age, gender and following correction for multiple comparisons. DRB1*14:54:01 (*n* = 5/137 vs. 0/350; OR = 28.592, FDR *p* = 0.004) and DRB1*14:24:01 (*n* = 4/137 vs. 0/350; OR = 23.307, FDR *p* = 0.010) were detected only in cases (Table 4). In contrast, DRB1*14:01:01 was absent in cases (*n* = 0/137 vs. 31/350; OR = 0.039, FDR *p* = 0.004), suggesting a protective effect. The initially significant associations of DRB1*11:04:01 (*p* = 0.029) and DRB1*04:03:01 (*p* = 0.050) with altered risk of ALL did not remain significant after correction for multiple comparisons (Table 4). All other DRB1 alleles showed no statistically significant differences even before Bonferroni adjustment (Table 4).

The most notable associations were seen with the HLA‐DQA1 locus, where 17 alleles were identified, 13 of which were common. DQA1*05:05:01 (*n* = 32/137 vs. 0/350; OR = 210.312), DQA1*03:03:01 (*n* = 19/137 vs. 0/350; OR = 119.039), DQA1*01:04:01 (*n* = 10/137 vs. 0/350; OR = 61.679), DQA1*03:02:01 (*n* = 10/137 vs. 0/350; OR = 61.679) and DQA1*05:03:01 (*n* = 7/137 vs. 0/350; OR = 43.509) were absent in controls and significantly more frequent in patients (all at FDR *p* < 0.0001) (Table 5). Significant negative associations were observed with DQA1*03:01:01 (*n* = 16/137 vs. 146/350; OR = 0.262), DQA1*01:01:01 (*n* = 14/137 vs. 110/350; OR = 0.321) and DQA1*05:01:01 (*n* = 269/137 vs. 155/350; OR = 0.412) (all at FDR *p* < 0.0001), which were underrepresented in patients and associated with a lower risk of ALL (Table 5). The large ORs observed for select DQA1 alleles reflect their absence in controls and their low absolute frequencies. As such, they should be considered as indicative of enrichment rather than precise risk quantification.

A total of 18 HLA‐DQB1 alleles were identified, of which 15 were common. Of these, DQB1*02:02:01 was detected exclusively in cases (*n* = 30/137 vs. 0/350; OR = 174.767, FDR *p* < 0.001), while DQB1*02:01:01 significantly reduced in cases (*n* = 28/137 vs. 148/350; OR = 0.425, FDR *p* = 0.001). While DQB1*05:03:01 was initially significantly associated with a reduced risk of ALL (*p* = 0.046), this association was no longer significant after adjustment (Table 6). None of the remaining alleles was linked with an altered risk of ALL.

Conversely, no significant links were found between DPB1 alleles and ALL risk. Several alleles exhibited nominal frequency differences, but none remained significant after correcting for multiple testing. Effect estimates were modest with broad CIs, especially for low‐frequency alleles, suggesting limited precision. (Table 7).

Collectively, this indicates that alleles found only in cases or controls must be viewed in the context of regional HLA variation. Protective alleles detected solely in controls, such as A*31:01:01 and C*04:01:02, are known in Central and East Asian populations, suggesting disease‐related depletion rather than true exclusivity. In contrast, risk alleles observed only in cases may reflect population‐specific effects driven by local linkage disequilibrium, drift or selection, or they may simply tag nearby causal variants within the MHC.

### 3.3 Sensitivity Analysis of HLA Associations Stratified by Allele Frequency (≥ 1% Threshold)

While multiple alleles demonstrated nominal associations, only a limited subset remained significant after multiple testing correction, indicating that most signals should be interpreted with caution. Nominal associations, particularly those involving low‐frequency alleles, should be considered hypothesis‐generating and require independent validation. Several associations persisted after restricting analyses to alleles with frequencies ≥ 1%, with A*31:01:01 showing a stable protective effect and B*51:01:01 and B*44:02:01 remaining enriched in cases (Table S2). Most signals at HLA‐C, DRB1, DQA1 and DQB1 disappeared under this threshold, indicating that many large effect estimates were driven by rare or group‐exclusive alleles rather than common‐variant associations.

### 3.4 Haplotype Frequency Distribution

**FIGURE 1:**
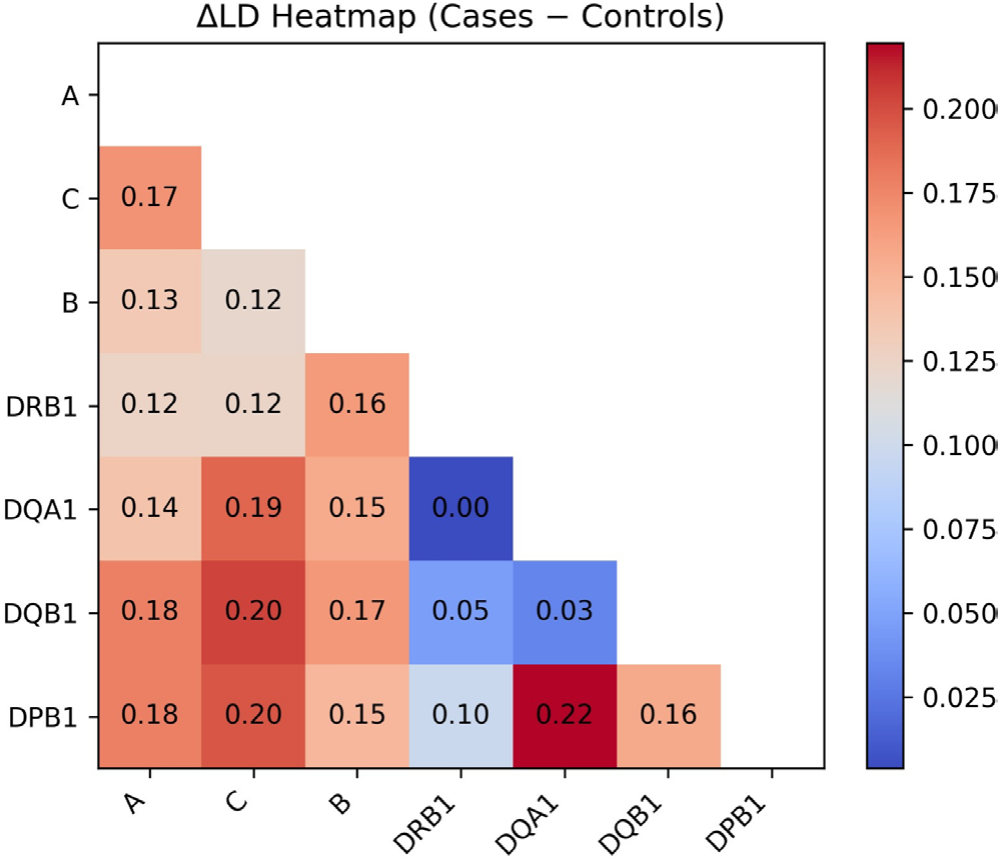
Differential linkage disequilibrium (ΔLD) across HLA Loci. This lower‐triangular heatmap displays the pairwise difference in Linkage Disequilibrium (ΔLD) between the Case and Control cohorts, quantified using the squared correlation coefficient (*r* ^2^) derived from EM‐based haplotype estimates (ΔLD = *r* ^2^_cases − *r* ^2^_controls). The colour gradient represents the magnitude and direction of the shift: Warm red tones indicate higher LD (*r* ^2^) in cases, while cool blue tones indicate higher LD in controls. The numerical values within cells represent the specific Δ*r* ^2^ for each locus pair.

A structured, locus‐specific shift in LD across Class I and Class II loci was noted using ΔLD heatmap analysis based on composite multiallelic *r* ^2^ estimates. Although several locus pairs showed higher LD in cases, these differences may reflect both enrichment of disease‐related haplotypes and the influence of sample size and haplotype diversity. Class I deviations comprised HLA‐A–HLA‐C (0.17), HLA‐A–HLA‐B (0.13) and HLA‐C–HLA‐B (0.12), which reflected altered allelic co‐inheritance (Figure 1). The strongest ΔLD values among Class II loci were DQA1–HLA‐C (0.19), DQB1–HLA‐C (0.20) and DPB1–DQA1 (0.22) (Figure 1). Additional elevated signals appeared for DQB1–HLA‐A (0.18), DPB1–HLA‐A (0.18) and DPB1–HLA‐C (0.20). DPB1–DRB1 (0.10) and DQB1–DRB1 (0.05) showed intermediate differences, while DQA1–DRB1 showed minimal deviation (0.00) (Figure 1).

**FIGURE 2:**
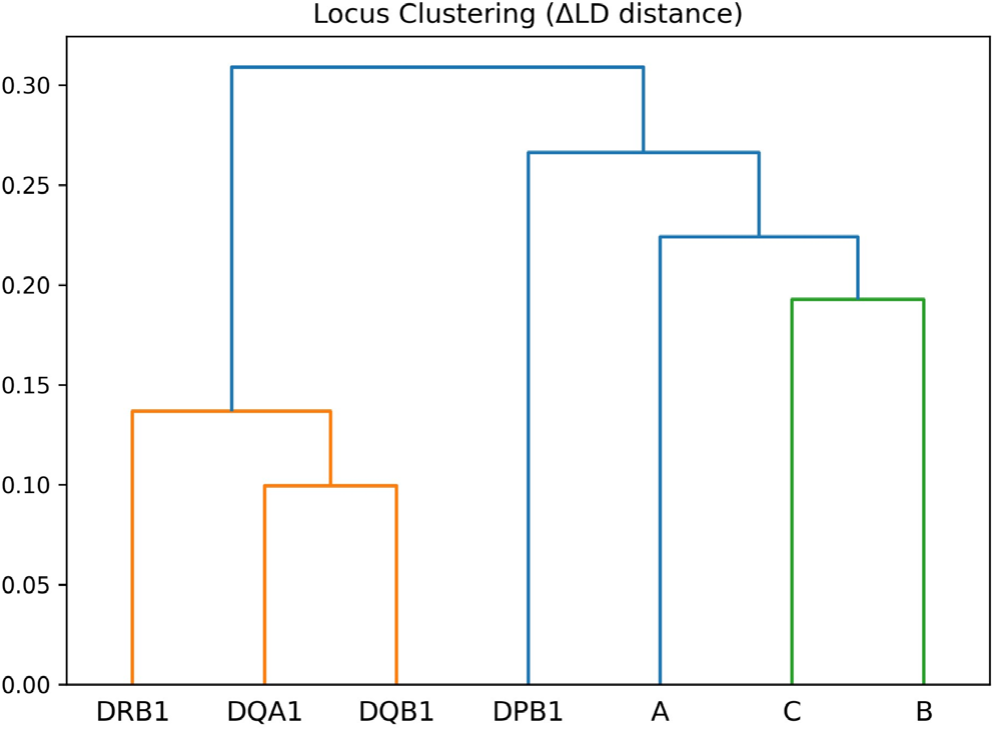
ΔLD hierarchical clustering. Clustering of ΔLD distances showed tight grouping of DQA1–DQB1, with DRB1 joining at a slightly higher distance, reflecting strong cohesion within the core Class II block. DPB1 entered only at a much larger distance, indicating weaker coupling. For Class I, HLA‐B and HLA‐C clustered closely, with HLA‐A joining at a greater distance. At the top level, Class I and Class II formed clearly separated clusters, indicating preserved class‐specific structure despite intra‐class ΔLD shifts.

Based on the change in LD (ΔLD) distance, calculated as ΔLD = *r* ^2^_cases_ − *r* ^2^_controls_, hierarchical clustering demonstrated unique structural groups (Figure 2). These comprised DQA1 and DQB1, which formed the closest pair, with DRB1 joining at a higher distance, thereby consistent with the tightly integrated organisation of the core Class II block. As expected, DPB1 remained separate and entered the hierarchy at a greater distance, consistent with its weaker connection to DRB1–DQA1–DQB1. Among Class I loci, HLA‐B and HLA‐C clustered closely, while HLA‐A joined at a greater distance, indicating more divergent ΔLD relationships involving HLA‐A. At the highest level, Class I (A, B, C) and Class II (DRB1, DQA1, DQB1, DPB1) segregated clearly, demonstrating that disease‐associated LD perturbations preserved major class‐specific architecture while altering intra‐class structure (Figure 2).

Class I haplotype analysis showed selective differences between ALL cases and controls (Table 8). The haplotype A*33:03:01~C*03:02:02~B*58:01:01 was markedly enriched in cases and demonstrated a strong association (*n* = 12/137 vs. 0/350; *χ* ^2^ = 31.039, *p* < 0.0001), which remained significant after Bonferroni and BH‐FDR correction (both < 0.001) and showed a large effect size (OR = 66.714, 95% CI: 3.936–1130.853). The wide CIs associated with this estimate reflect the low haplotype frequency and limited sample size, suggesting reduced precision of the effect estimate despite statistical significance. In contrast, A*33:03:01~C*03:02:01~B*58:01:01 appeared only in controls, and showed a positive association (*n* = 0/137 vs. 25/350; *p* = 0.002), which persisted after Bonferroni correction [*p* = 0.018; FDR = 0.009; OR (95% CI) = 0.048 (0.003–0.795)]. A*30:01:01~C*06:02:01~B*13:02:01 and A*02:01:01~C*07:02:01~B*07:02:01 haplotypes showed nominal associations (*p* = 0.060 and 0.042), which did not withstand correction.

**TABLE 8:** Class I (A~C~B) haplotypes associated with acute lymphoblastic leukaemia.

Haplotype	All^a^	Cases^a^	Controls^a^	Chi square	*p* ^b^	Corrected *p* ^c^	FDR (wald *p*)^d^	OR (95% CI)^e^
33:03:01~03:02:02~58:01:01	0.01232	12 (0.04380)	0 (0.00000)	31.039	< 0.0001	< 0.001	< 0.001	66.714 (3.936–1130.853)
33:03:01~03:02:01~58:01:01	0.02560	0 (0.00000)	25 (0.03571)	10.044	0.002	0.018	0.009	0.048 (0.003–0.795)
30:01:01~06:02:01~13:02:01	0.01951	9 (0.03285)	10 (0.01429)	3.547	0.060	0.716	0.119	2.343 (0.942–5.831)
02:01:01~07:02:01~07:02:01	0.01098	7 (0.02536)	4 (0.00617)	4.139	0.042	0.503	0.119	2.891 (0.810–10.320)
02:07:01~01:02:01~46:01:01	0.01185	0 (0.00000)	10 (0.01494)	4.139	0.042	0.503	0.119	0.115 (0.007–1.959)
02:01:01~06:02:01~13:02:01	0.01834	2 (0.00730)	15 (0.02073)	2.131	0.144	1.000	0.217	0.347 (0.079–1.534)
01:01:01~07:01:01~08:01:01	0.01022	1 (0.00365)	9 (0.01286)	1.643	0.199	1.000	0.267	0.281 (0.035–2.230)
02:01:01~03:04:01~40:02:01	0.01148	5 (0.01825)	4 (0.00637)	2.887	0.089	1.000	0.153	2.898 (0.802–10.468)
24:02:01~06:02:01~13:02:01	0.01218	2 (0.00730)	10 (0.01382)	0.707	0.400	1.000	0.481	0.525 (0.114–2.421)
24:02:01~03:04:01~40:02:01	0.02120	7 (0.02555)	14 (0.01954)	0.342	0.558	1.000	0.609	1.316 (0.523–3.307)
03:01:01~07:02:01~07:02:01	0.01236	0 (0.00000)	10 (0.01362)	3.768	0.052	0.627	0.119	0.643 (0.151–2.736)
24:02:01~07:02:01~07:02:01	0.02127	5 (0.01825)	16 (0.02338)	0.242	0.623	1.000	0.623	0.776 (0.282–2.134)

^a^ Haplotype counts (frequencies); haplotypes estimated using the expectation–maximization (EM) algorithm.

^b^ Adjusted for age and gender.

^c^ Denotes the Bonferroni‐adjusted age‐ and sex‐adjusted *p* value derived from logistic regression, calculated as: $p_{c}=1-{\left(1-p_{\mathrm{adj}}\right)}^{n}$, where n is the number of alleles tested at that locus.

^d^ FDR = false discovery rate, calculated according to Benjamini–Hochberg (BH) Procedure. Associations surviving both Bonferroni and FDR correction were considered robust, whereas those significant only under FDR were treated as suggestive due to their less stringent error control.

^e^ Odds ratios (ORs) and 95% confidence intervals (CIs) obtained from univariate logistic regression models.

Class II haplotype analysis demonstrated selective differences between ALL cases and controls (Table 9). Haplotypes DRB1*07:01:01~DQA1*01:02:01~DQB1*02:02:01~DPB1*04:01:01 (*n* = 4/137 vs. 0/350) and DRB1*15:01:01~DQA1*05:05:01~DQB1*06:02:01~DPB1*04:02:01 (*n* = 4/137 vs. 0/350) were seen exclusively in cases, showing strong associations (*p* = 0.001) that remained significant after Bonferroni correction (*p* = 0.009) and BH‐FDR adjustment (*q* = 0.005), with large effect sizes [OR (95% CI) = 25.785 (1.383–480.691)]. Similarly, DRB1*11:04:01~DQA1*05:05:01~DQB1*03:01:01~DPB1*04:01:01 (*n* = 3/137 vs. 0/350), DRB1*07:01:01~DQA1*02:01:01~DQB1*02:02:01~DPB1*04:01:01 (*n* = 3/137 vs. 0/350) and DRB1*07:01:01~DQA1*02:01:01~DQB1*02:02:01~DPB1*02:01:02 (*n* = 3/137 vs. 0/350) were detected only in cases, remaining significant after correction (*p* = 0.004; Bonferroni *p* = 0.043; FDR *q* = 0.009). A few Class II haplotypes appeared only in controls, but none remained significant after correction. Several showed wide CIs due to sparse counts and limited precision, and low‐frequency haplotypes carried added uncertainty because EM‐based inference is unstable at small counts; these findings were therefore interpreted cautiously.

**TABLE 9:** Class II (DRB1~DQA1~DQB1~DPB1) haplotypes associated with acute lymphoblastic leukaemia.

Haplotype	Cases^a^	Controls^a^	Chi‐square	*p* ^b^	Corrected *p* ^c^	FDR (BH)^d^	OR (95% CI)^e^
07:01:01~01:02:01~02:02:01~04:01:01	4 (0.01610)	0 (0.00000)	11.338	0.001	0.009	0.005	25.785 (1.383–480.691)
15:01:01~05:05:01~06:02:01~04:02:01	4 (0.01610)	0 (0.00000)	11.338	0.001	0.009	0.005	25.785 (1.383–480.691)
11:04:01~05:05:01~03:01:01~04:01:01	3 (0.01210)	0 (0.00000)	8.495	0.004	0.043	0.009	19.974 (1.028–388.080)
07:01:01~02:01:01~02:02:01~04:01:01	3 (0.01210)	0 (0.00000)	8.495	0.004	0.043	0.009	19.974 (1.028–388.080)
07:01:01~02:01:01~02:02:01~02:01:02	3 (0.01210)	0 (0.00000)	8.495	0.004	0.043	0.009	19.974 (1.028–388.080)
03:01:01~01:02:01~02:01:01~04:01:01	3 (0.01210)	1 (0.00140)	4.960	0.026	0.311	0.052	8.559 (0.886–82.673)
07:01:01~01:02:01~02:01:01~04:01:01	0 (0.00000)	9 (0.01290)	3.219	0.073	0.873	0.125	0.146 (0.008–2.526)
03:01:01~05:01:01~02:01:01~04:01:01	4 (0.01610)	7 (0.01000)	0.599	0.439	1.0	0.64	1.623 (0.471–5.592)
01:01:01~01:01:01~05:01:01~02:01:02	2 (0.00810)	3 (0.00430)	0.498	0.48	1.0	0.64	1.889 (0.314–11.372)
01:01:01~01:01:01~05:01:01~04:01:01	2 (0.00810)	9 (0.01290)	0.367	0.545	1.0	0.654	0.624 (0.134–2.909)
03:01:01~01:03:01~02:01:01~04:01:01	3 (0.01210)	7 (0.01000)	0.077	0.781	1.0	0.852	1.212 (0.311–4.725)
15:01:01~01:02:01~06:02:01~04:01:01	3 (0.01210)	8 (0.01140)	0.007	0.933	1.0	0.933	1.059 (0.279–4.025)

^a^ Haplotype counts (frequencies); haplotypes estimated using the expectation–maximization (EM) algorithm.

^b^ Adjusted for age and gender.

^c^ Denotes the Bonferroni‐adjusted age‐ and sex‐adjusted *p* value derived from logistic regression, calculated as: $p_{c}=1-{\left(1-p_{\mathrm{adj}}\right)}^{n}$, where n is the number of alleles tested at that locus.

^d^ FDR = false discovery rate, calculated according to Benjamini–Hochberg (BH) Procedure. Associations surviving both Bonferroni and FDR correction were considered robust, whereas those significant only under FDR were treated as suggestive due to their less stringent error control.

^e^ Odds ratios (ORs) and 95% confidence intervals (CIs) obtained from univariate logistic regression models.

## 4 Discussion

This study provides a comprehensive high‐resolution NGS characterisation of HLA Class I and Class II allele distributions, haplotypes and LD structure in ALL among ethnic Kazakhs. This extends related studies in Central Asia on the use of HLA genotyping to interpret HLA‐based susceptibility in hematologic malignancies [14, 29, 30]. A key finding of this study was the strong associations of DRB1, DQA1 and DQB1 alleles with altered risk of ALL, consistent with the role of Class II molecules in adaptive immunity via regulation of CD4^+^ T‐cell activation and antigen presentation [17, 31]. In contrast to their lack of association with aplastic anaemia [29] and AML [30], we now report that Class I alleles, particularly those at the HLA‐C and HLA‐B loci, are associated with an altered risk of ALL. The presence of both positive and negative associations across loci in this study contrasts with the patterns observed in AA and AML, where Class I effects were more prominent, and Class II bias was less evident in the same ethnic background [29, 30]. This suggests that Class I and Class II alleles and haplotypes may play a more integrated and broader role in ALL pathogenesis [32].

Unusually large ORs for several low‐frequency HLA alleles are credible given that most loci met HWE expectations in controls, with only mild departures noted for DQA1 and DPB1. These deviations reflect complex allele frequencies, modest sample size or minor population structure, more than genotyping error or major stratification [18, 33]. These patterns support the view that rare, high‐impact variants can become enriched in disease states when high‐resolution typing captures their full specificity [34]. Post hoc power analysis shows that the study is well powered for moderate effects among common alleles but has limited ability to detect modest associations for rare variants, which explains the exaggerated estimates observed for low‐frequency alleles and haplotypes. Rare‐allele signals likely reflect a mix of true enrichment and imprecision, and several associations arose from alleles present only in cases or controls, producing large ORs driven by zero‐cell comparisons. Even with Haldane–Anscombe and Firth corrections, these estimates remain highly sensitive to sparse data, and wide CIs in the ≥ 1% analysis reinforce that such findings are provisional and require replication in larger cohorts.

ALL comprises diverse cytogenetic and molecular subtypes, including lesions such as BCR‐ABL1 and KMT2A rearrangements, which differ across paediatric and adult disease. Because analyses were not stratified by age or subtype, the reported associations should be interpreted cautiously, as they reflect combined effects across a heterogeneous spectrum and may not apply uniformly to specific molecular or immunophenotypic subgroups. The extensive HLA polymorphism observed suggests that uncommon alleles may influence peptide binding differently from common variants, consistent with the strong associations noted with rare DQA1 alleles [18]. The contrasting behaviour of DPB1*04:01:01, neutral in ALL yet protective in AML [21, 30], illustrates disease‐dependent effects across loci [35]. Replication and functional studies remain essential despite statistical robustness [33, 35], and very large ORs are interpreted as markers of informative rare haplotypic backgrounds rather than precise effect sizes. While the presence of rare alleles exclusively in cases does not necessarily imply causality, it may reflect population‐specific enrichment, LD with causal variants or stochastic variation.

Emerging evidence indicates that HLA allele and haplotype associations may vary across ALL subgroups defined by immunophenotype and cytogenetic or molecular features (e.g., B‐ALL vs. T‐ALL, or recurrent genomic alterations) [36, 37]. Accordingly, the associations observed here should be interpreted as aggregate effects across a heterogeneous cohort. Because complete subtype information was not available for all patients and subgroup sizes were small, stratified analyses were not feasible, leaving open the possibility that some signals are driven by specific ALL subtypes rather than representing uniform effects [38]. These interpretations assume accurate germline HLA inference, which is supported by the sequencing depth and quality control metrics applied in this study. Larger, well‐characterised cohorts with detailed immunophenotypic and molecular annotation will be needed to assess the consistency and subtype specificity of these associations.

Locus‐specific Class I associations were observed in ethnic Kazakhs with ALL. Aside from a corrected protective effect at A*31:01:01, no other HLA‐A alleles showed meaningful risk differences. In contrast, several HLA‐B and HLA‐C alleles were restricted to either cases (C*03:02:02, C*04:01:01, B*51:01:01, B*40:01:02) or controls (C*04:01:02, C*03:02:01, B*01:01:01, B*41:02:01), generating large ORs that remained significant after FDR correction. The stable genotype distribution in controls argues against technical artefacts and supports non‐random enrichment at these loci [39, 40]. Mechanistically, stronger B and C signals are compatible with a role for CD8^+^ T‐cell surveillance in ALL, given Class I involvement in presenting endogenous peptides [41]. While these findings suggest that lineage‐specific differences in antigen presentation may underlie the distinct HLA patterns observed in ALL, AML and aplastic anaemia [29, 40], this remains speculative in the absence of functional data. Because HLA‐C also modulates NK‐cell education, case‐enriched subtypes may influence NK responsiveness, complementing Class II–driven CD4^+^ pathways in ALL susceptibility.

Consistent with prior reports from other populations, HLA Class II associations with overall ALL risk vary substantially across ethnic groups, with notable differences in the involved variants differing across groups [20, 21]. This underscores the need for population‐specific analyses and suggests distinct immunogenetic patterns in Kazakhs. In this cohort, both at‐risk and protective Class II alleles were observed, with some variants restricted to either cases or controls. Despite the involvement of low‐frequency alleles, preservation of HWE in controls and persistence after FDR correction reduce concerns about technical bias [42]. DPB1 showed no significant effects, indicating that associations cluster along the DQ/DR axis. DQA1 remained the strongest contributor, with alleles such as DQA1*05:05:01 and DQA1*03:03:01 detected only in cases, consistent with effects of DQα polymorphisms on peptide binding [40]. Differences from United Kingdom and Iraqi cohorts [18, 25], together with broader European and East Asian findings [22, 23, 24], highlight marked population heterogeneity in HLA–ALL associations.

It was interesting to note that, while DQA1 also predominated in the AML and AA Kazakhstani cohorts [29, 30], the specific alleles varied between the diseases. This was demonstrated by the association of DQA1*05:01:01 in ALL and AA, but not in AML, and by the association of DQA1*01:02:01 and DQA1*01:03:01 with a decreased risk of AML, but not of AA or ALL [29, 30]. The observed LD patterns and haplotype analyses support the possibility that coordinated variation across Class II loci contributes to the detected associations, with DRB1*07:01:01∼DQA1*01:02:01∼DQB1*02:02:01∼DPB1*04:01:01 being linked to increased risk in ALL but showing no association with altered risk of either AA or AML [29, 30]. Although not directly evaluated in this study, the specific association of HLA markers with various haematological disorders may, in part, reflect variation in antigen presentation across haematopoietic lineages, potentially influenced by differences in peptide repertoires expressed by early lymphoid, myeloid and erythroid cells [16, 43]. Notably, sensitivity analyses limited to alleles with frequencies ≥ 1% showed that only A*31:01:01, B*51:01:01 and B*44:02:01 retained significant associations after correction, whereas many large‐effect signals (HLA‐C, DQA1) were not sustained, indicating dependence on rare variants and sparse counts (Table S2). These results underscore the need to distinguish robust common‐allele signals from exploratory associations driven by low‐frequency variation, necessitating validation through functional studies to confirm or, alternatively, rule out this interpretation.

Haplotype inference relied on EM applied to unphased genotypes, which yields dependable frequency estimates for common combinations but is less stable for rare haplotypes in regions with extensive allelic diversity, such as the MHC [23, 43]. As a result, estimates for low‐frequency haplotypes are more uncertain and were interpreted with appropriate caution [12, 28]. Multi‐locus haplotype analysis revealed associations not captured by single‐allele comparisons; however, several haplotypes were present at low frequencies, resulting in wide CIs and limited precision of effect estimates [44]. Haplotype A*33:03:01~C*03:02:02~B*58:01:01 was strongly associated with ALL, while the closely related A*33:03:01~C*03:02:01~B*58:01:01 was largely protective, underscoring the significance of minor HLA‐C variations. The large OR and broad CI reflect sparse observations, rather than a precisely quantified effect, and are inherent to high‐resolution, multi‐locus HLA analyses, where combinatorial diversity generates numerous low‐frequency haplotypes. The exclusive distribution of select Class II haplotypes in patients, including DRB1*07:01:01~DQA1*01:02:01~DQB1*02:02:01~DPB1*04:01:01, supports coordinated influences between DQ and DR [43]. It is also possible that these haplotypes act as proxies for underlying causal variants within the MHC, rather than representing independent functional units.

These findings align with ΔLD analyses, indicating structured LD shifts in haplotype assignment, particularly within Class II loci. While the changes in LD patterns can indicate enrichment or loss of rare extended haplotypes, they should be interpreted cautiously given the influence of sample size and allele frequency distributions. In the HLA region, where alleles segregate in tight linkage blocks, such differences complement single‐allele analyses by capturing multi‐locus variation that may contribute to disease susceptibility. Hierarchical clustering confirmed a strong DQA1–DQB1 linkage and a weaker connection with DPB1 [45]. The discrepancies among nearly identical haplotypes, and thus allele‐specific variations in peptide‐binding ranges [46], influence T‐cell activation and immune processes [47]. Furthermore, the likelihood of technical artefacts in associations involving low‐frequency haplotypes was accounted for using statistical corrections and control distributions [48]. The concordance between the ΔLD changes and haplotypic associations supports the notion that non‐random multi‐locus HLA structures influence ALL susceptibility [23, 42].

The higher LD in cases likely reflects both enrichment of disease‐related haplotypes and inflation from small sample size and uneven frequencies, suggesting differences in haplotype structure rather than selection, and, given MHC complexity and population effects, should be viewed cautiously and considered hypothesis‐generating. Also, ΔLD results should be interpreted cautiously, as differences may also arise from sampling variability, allele frequency differences, or population structure rather than true biological effects [12, 14]. Accordingly, ΔLD analysis should be viewed as a hypothesis‐generating approach that complements allele‐ and haplotype‐level association testing [23]. Collectively, these findings highlight the importance of considering haplotype‐level architecture when interpreting HLA‐disease associations [28, 43].

Consistent with earlier observations, Class II associations, particularly DQA1, were the most prominent, with the accompanying Class I associations providing complementary insight. Class I molecules present endogenous peptides to CD8^+^ T cells, whereas Class II molecules present exogenous peptides to CD4^+^ T cells [11, 17], and signals across both pathways suggest coordinated effects on immune priming and cytotoxic responses [23]. Significant alleles after correction included A*31:01:01, B*51:01:01 and B*44:02:01 alongside Class II variants, indicating that risk is unlikely to map to a single arm of antigen presentation [11, 16]. Mechanistically, HLA polymorphism may influence leukaemogenesis through altered peptide‐binding repertoires, infection‐related pathways implicated in childhood ALL [21], and differences in thymic selection shaping T‐cell surveillance. These processes support the predominance of Class II signals and align with broader immunogenetic patterns shared across ALL, AML and AA, with LD remodelling highlighting targets for future functional studies. In the absence of functional validation, these mechanisms remain speculative but provide a testable framework for future studies.

The findings could have clinical significance for HSCT and disease risk assessment [16]. Population‐specific HLA allele and haplotype patterns may guide donor‐matching strategies for Kazakh patients, particularly when certain patient haplotypes influence the chance of finding matches within regional registries [14, 16]. Mechanistically, differences in HLA‐mediated antigen presentation might alter immune recognition of leukaemic cells and affect post‐transplant responses, including graft‐versus‐leukaemia effects, offering a biological explanation for the observed associations [12, 14]. The links between specific HLA variants and susceptibility to ALL also suggest that HLA typing could improve existing clinical, cytogenetic and molecular markers for risk stratification [19, 20, 30]. Furthermore, since biologically distinct B‐ and T‐cell ALL subtypes may differentially influence HLA associations, and as subtype stratification was not possible, the observed signals should be interpreted as a composite across lineages rather than reflecting subtype‐specific effects. This suggests that any potential clinical application remains exploratory and requires validation in larger, independent cohorts.

This study has several strengths, including high‐resolution NGS analysis of multiple HLA loci in ALL among ethnic Kazakhs, thus addressing a major geographic and population gap [49, 50]. In addition, comprehensive genotyping of the seven HLA loci, uniform genotyping workflows, and adopted quality control measures (HWE testing, age‐ and sex‐adjusted logistic regression and Bonferroni and BH‐FDR correction) enhanced analytic reliability [51]. Bonferroni identifies high‐confidence signals, while FDR offers sensitivity in correlated tests. Alleles significant only under FDR are considered suggestive and reported transparently but require cautious interpretation and independent replication. Furthermore, the inclusion of self‐declared native Kazakhs in the patient and control groups minimised population stratification, while allele‐, haplotype‐ and ΔLD‐based analyses provided higher resolution compared to single‐marker testing. The strongest Class II haplotype signal and opposing Class I haplotypes differing only at the third field of HLA‐C underscore how subtle allelic differences can markedly alter risk [23, 44], reinforcing the value of high‐resolution typing and the need for integrated fine‐mapping approaches incorporating SNPs, amino acid polymorphisms and regulatory annotations [50]. While using stem cell donors as controls offers a well‐screened, disease‐free comparison group, it may introduce selection bias, as donors are typically healthier than the general population. Although their allele frequencies closely matched published Kazakh data, suggesting representativeness, we cannot fully rule out some degree of selection bias.

Several limitations are also noteworthy. The limited sample size reduced power for rare alleles, yielding unstable estimates even with Haldane–Anscombe and Firth adjustments and zero‐cell comparisons required continuity corrections that may distort effect sizes. Departures from HWE at DQA1 and DPB1 likely reflect population structure or allelic complexity rather than technical error but still warrant caution. Post hoc power analyses indicate low sensitivity to modest effects among rare alleles and haplotypes, increasing the risk of false negatives and inflated associations. Although control frequencies align with external datasets, donor‐registry recruitment and the retrospective design introduce potential selection bias and residual confounding. The influence of somatic alterations in leukaemic blasts, including LOH in the HLA region, particularly in samples with high blast content, is an added limitation, as genotyping was performed on diagnostic blood without consistent blast fraction data. As such, some effect on genotype inference cannot be excluded. The stable call rates, absence of allelic imbalance, high sequencing depth and concordance with known Kazakh allele frequencies support the reliability of the results. The absence of age and indication stratification limits interpretation and EM‐based haplotype inference adds uncertainty for rare combinations. Restricting analyses to ethnic Kazakhs limits generalisability, consistent with evidence that HLA–ALL associations are highly context‐dependent across populations [12, 16, 21].

## 5 Conclusion

This study provides a high‐resolution characterisation of HLA Class I and II variation in a mixed paediatric–adult cohort of ethnic Kazakh patients with ALL, providing a foundation for future work that incorporates HLA diversity into donor‐selection strategies and risk‐stratification models for Central Asian populations. It established population‐specific allele and haplotype associations dominated by Class II signals centred on the DRB1–DQA1–DQB1 axis, alongside additional Class I signals at HLA‐B and HLA‐C. Although no significant allele‐level associations were identified at the DPB1 locus, LD patterns suggest potential involvement within extended haplotypes, with shifts in LD structure indicating a contribution of DPB1 haplotype architecture. However, these findings are indirect, warranting fine‐mapping and thus should be interpreted cautiously. These findings suggest that both HLA Class I and Class II pathways contribute to ALL susceptibility, with Class II signals predominating and Class I associations providing complementary evidence of altered immune recognition, collectively influencing leukemogenic risk. Given the known heterogeneity of ALL, these associations should be interpreted as reflecting aggregate effects across mixed disease subtypes. Shared disease‐specific patterns across ALL, AML and AA point to a broader immunogenetic framework shaped by peptide binding and immune surveillance, while structured LD remodelling highlights targets for future functional studies.

Although several associations show notable effect sizes, interpretation remains cautious due to extensive polymorphism and frequency constraints, with several associations driven by low‐frequency alleles and not persisting after applying a ≥ 1% frequency threshold. While results are supported by HWE conformity and multiple‐testing correction, they still require independent replication and functional validation. Also, the presence of sparse cells, including alleles and haplotypes observed exclusively in one group, may inflate effect estimates despite statistical correction, limiting the precision of some associations. Collectively, these findings lay a foundation for population‐specific immunogenetic research in Central Asia with implications for risk modelling, donor matching and mechanistic studies of HLA‐driven leukemogenesis. Given the modest sample size and rarity of several alleles, these findings require confirmation. Larger, balanced cohorts with age, cytogenetic and treatment‐indication stratification and population‐based reference panels are needed to determine whether observed associations and LD patterns reflect true biology rather than sampling artefacts.

## Author Contributions

A.T. and W.Y.A. designed the study, analysed the data and wrote the manuscript. Z.B. assisted in interpreting the results. Z.Z., D.B., Z.S. and D.K. performed the analysis and collected data. S.A. and Z.M. are responsible for project administration. W.Y.A. supervised the study and finalised the manuscript. All authors reviewed the final version of the manuscript.

## Funding

The authors have nothing to report.

## Ethics Statement

This study was conducted in accordance with the principles of the Declaration of Helsinki II and was approved by the Local Commission on Bioethics of the Scientific and Production Center for Transfusiology on November 24, 2024 (SPCT/2024/6). Written informed consent was obtained from all adult participants prior to enrollment. For participants under the age of 18 years, written informed consent was obtained from their parent(s) or legal guardian(s), in accordance with institutional guidelines and applicable regulations.

## Conflicts of Interest

The authors declare no conflicts of interest.

## Supporting information


**Table S1:** Hardy–Weinberg equilibrium calculation.

**Table S2:** Summary of robust HLA associations after multiple‐testing correction and sensitivity analysis.

**Figure S1:** Linkage disequilibrium structure across HLA loci in controls and ALL cases. Heatmaps summarise pairwise LD between Class I (A, C, B) and Class II (DRB1, DQA1, DQB1, DPB1) loci using the squared correlation coefficient (*r* ^2^) derived from EM‐based haplotype estimates, with darker shading indicating stronger LD. Panel (A), representing controls, shows the expected MHC structure, including strong Class II coupling and moderate Class I linkage. Panel (B), representing ALL cases, displays uniformly elevated LD and strengthened cross‐class connections. Overall, the pattern suggests LD intensification and remodelling in ALL, reflecting altered haplotypic architecture rather than isolated allele effects.

## Data Availability

The data that support the findings of this study are available from the Mendeley Data Repository at: Almawi, Wassim (2026), ‘HLA Association with ALL’, Mendeley Data, V1, https://doi.org/10.17632/2wzpk42ycb.1.
