# Supplementary material for: High‐Resolution NGS HLA Typing Identifies Specific Class II–Dominant Risk Haplotypes and HLA LD Structure in Acute Lymphoblastic Leukaemia Among Ethnic Kazakhs
Source: HLA. 2026 Jun 9;107(6):e70791. doi: 10.1111/tan.70791 (PMC13250375; doi:10.1111/tan.70791)
Supplement: Supplementary file 1 — Table S1: Hardy–Weinberg equilibrium calculation. [file TAN-107-e70791-s001.docx]

**Supplementary Table 1**

**Hardy-Weinberg Equilibrium Calculation**

| **Locus** | **Alleles** | **df** | **HWE p value** | **Genotypes** | **df** | **HWE p value** |
| --- | --- | --- | --- | --- | --- | --- |
| A | 30 | 435 | 0.143 | 23 | 253 | 0.985 |
| C | 32 | 253 | 0.558 | 24 | 276 | 0.082 |
| B | 66 | 2145 | 0.998 | 54 | 1431 | 1.000 |
| DRB1 | 40 | 780 | 0.318 | 34 | 561 | 1.000 |
| DQA1 | 16 | 120 | 0.263 | 9 | 36 | 0.039 |
| DQB1 | 16 | 120 | 0.100 | 18 | 153 | 0.843 |
| DPB1 | 24 | 276 | 0.780 | 8 | 28 | 0.006 |
