# Supplementary material for: High‐Resolution NGS HLA Typing Identifies Specific Class II–Dominant Risk Haplotypes and HLA LD Structure in Acute Lymphoblastic Leukaemia Among Ethnic Kazakhs
Source: HLA. 2026 Jun 9;107(6):e70791. doi: 10.1111/tan.70791 (PMC13250375; doi:10.1111/tan.70791)
Supplement: Supplementary file 2 — Table S2: Summary of robust HLA associations after multiple‐testing correction and sensitivity analysis. [file TAN-107-e70791-s003.docx]

**Supplementary Table 2**

**Summary of Robust HLA Associations After Multiple-Testing Correction and Sensitivity Analysis**

| **Locus** | **Allele** | **Direction** | **OR (95% CI)** | **Bonferroni p** | **FDR p** | **≥1% Robust** |
| --- | --- | --- | --- | --- | --- | --- |
| HLA-A | A*31:01:01 | Protective | 0.037 (0.002–0.614) | 0.006 | 0.006 | Yes |
| HLA-B | B*51:01:01 | Risk | 6.289 (3.049–12.969) | <0.001 | <0.001 | Yes |
| HLA-B | B*44:02:01 | Risk | 5.265 (1.783–15.548) | 0.026 | 0.005 | Yes |
