# Supplementary figures and images for: High‐Resolution NGS HLA Typing Identifies Specific Class II–Dominant Risk Haplotypes and HLA LD Structure in Acute Lymphoblastic Leukaemia Among Ethnic Kazakhs
Source: HLA. 2026 Jun 9;107(6):e70791. doi: 10.1111/tan.70791 (PMC13250375; doi:10.1111/tan.70791)

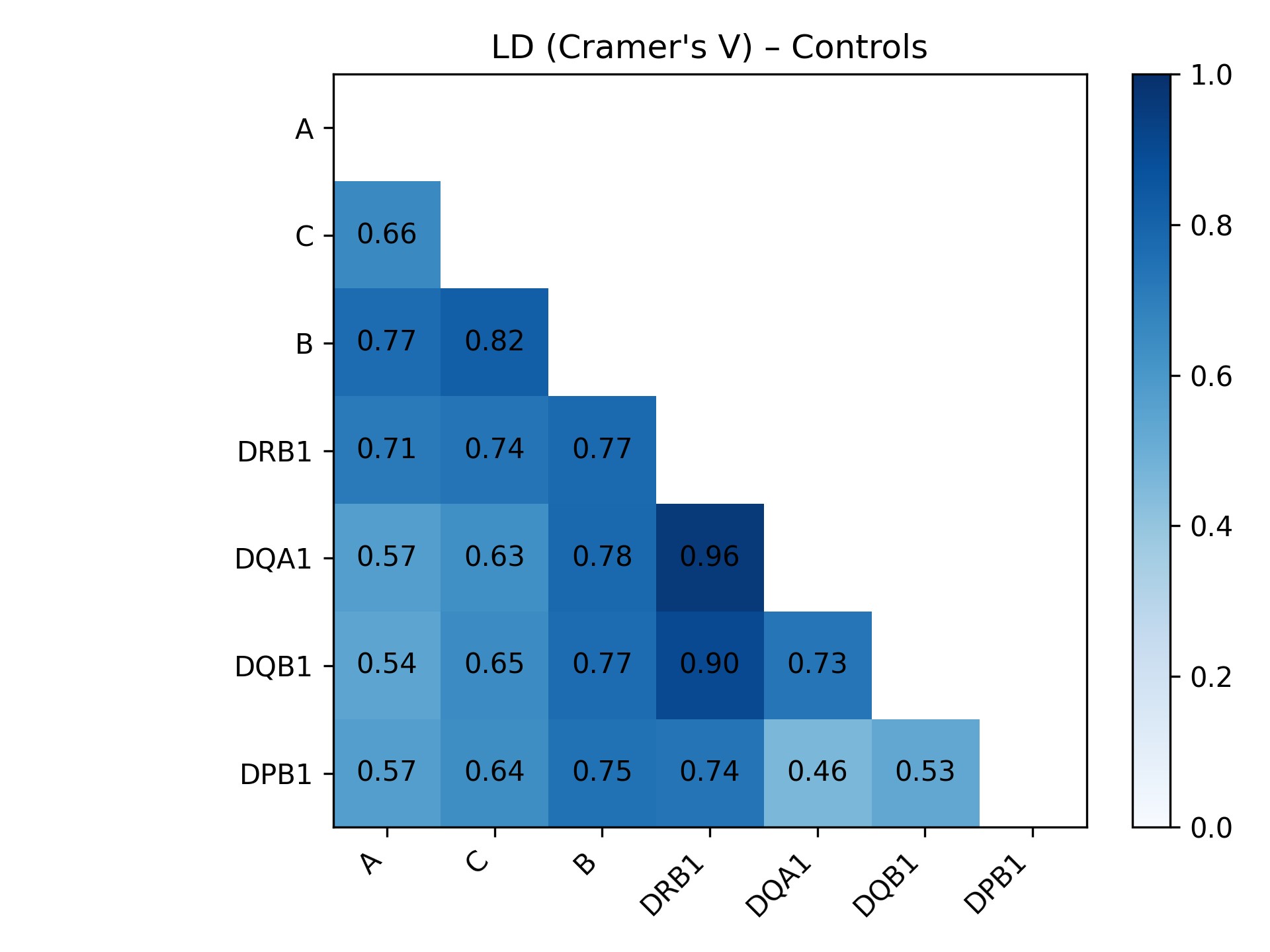

Supplement: Supplementary file 3 — Figure S1: Linkage disequilibrium structure across HLA loci in controls and ALL cases. Heatmaps summarise pairwise LD between Class I (A, C, B) and Class II (DRB1, DQA1, DQB1, DPB1) loci using the squared correlation coefficient (r 2) derived from EM‐based haplotype estimates, with darker shading indicating stronger LD. Panel (A), representing controls, shows the expected MHC structure, including strong Class II coupling and moderate Class I linkage. Panel (B), representing ALL cases, displays uniformly elevated LD and strengthened cross‐class connections. Overall, the pattern suggests LD intensification and remodelling in ALL, reflecting altered haplotypic architecture rather than isolated allele effects. [file TAN-107-e70791-s002.zip › tan70791-sup-0003-FigureS1@Supplementary Fig. 1A.jpg]

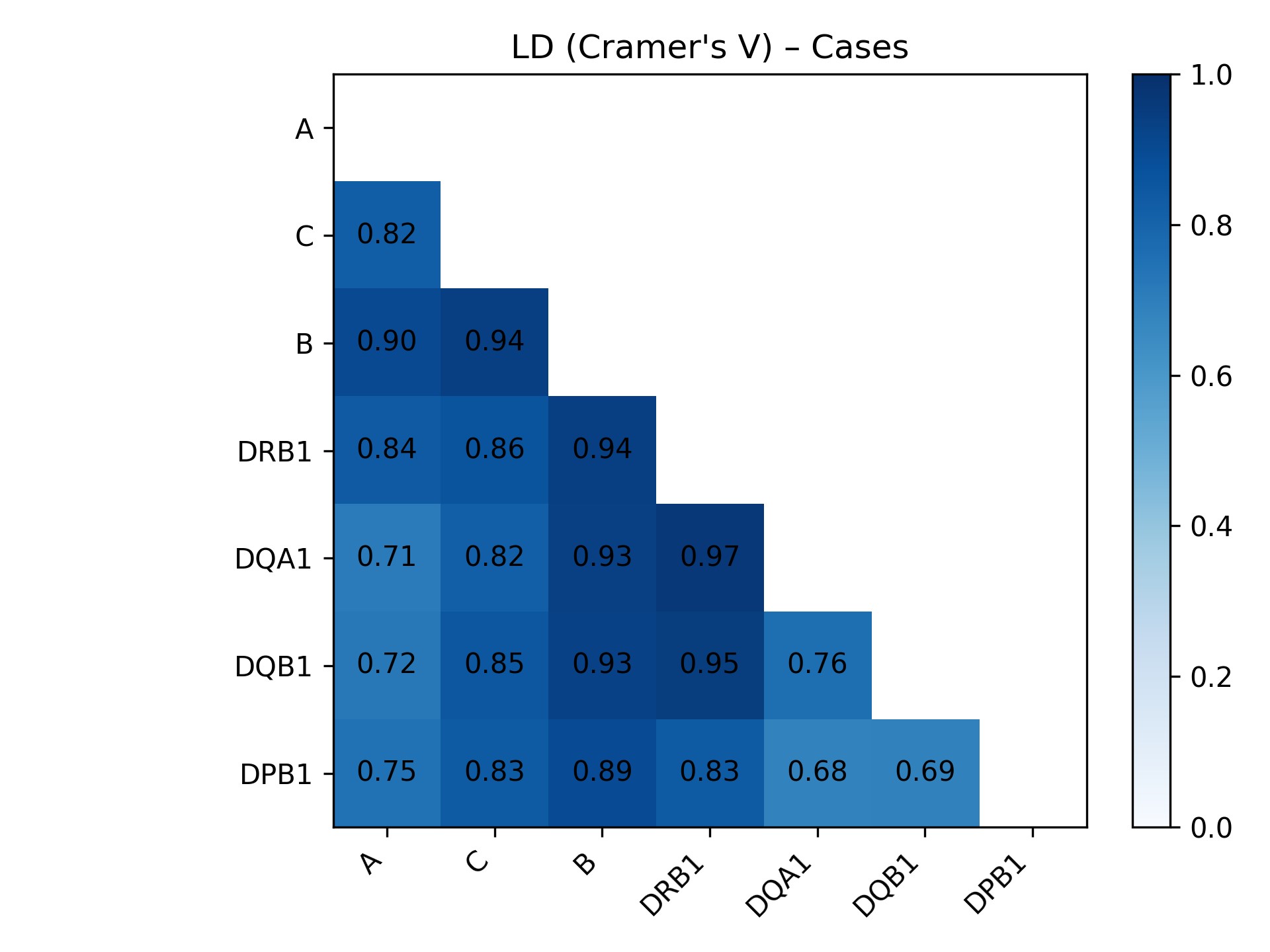

Supplement: Supplementary file 3 — Figure S1: Linkage disequilibrium structure across HLA loci in controls and ALL cases. Heatmaps summarise pairwise LD between Class I (A, C, B) and Class II (DRB1, DQA1, DQB1, DPB1) loci using the squared correlation coefficient (r 2) derived from EM‐based haplotype estimates, with darker shading indicating stronger LD. Panel (A), representing controls, shows the expected MHC structure, including strong Class II coupling and moderate Class I linkage. Panel (B), representing ALL cases, displays uniformly elevated LD and strengthened cross‐class connections. Overall, the pattern suggests LD intensification and remodelling in ALL, reflecting altered haplotypic architecture rather than isolated allele effects. [file TAN-107-e70791-s002.zip › tan70791-sup-0004-FigureS1@Supplementary Fig. 1B.jpg]
